# Supplementary material for: Identification and characterisation of serotonin signalling in the potato cyst nematode Globodera pallida reveals new targets for crop protection
Source: PLoS Pathog. 2020 Oct 2;16(10):e1008884. doi: 10.1371/journal.ppat.1008884 (PMC7556481; doi:10.1371/journal.ppat.1008884)
Supplement: S1 Fig — UNIPROT, CLUSTAL Omega program, accessed on 08/08/2014. Identity between C. elegans and G. pallida (cloned based on gene model GPLIN_000654600) CAT-1a is 51.8%, similar positions 95. *—identical positions,: and.—similar positions. (DOCX) [file ppat.1008884.s001.docx]

**Supplementary Figure 1. Alignment of amino acid sequences of *G. pallida* (gp) and *C. elegans* (ce) CAT-1.** UNIPROT, CLUSTAL Omega program, accessed on 08/08/2014. Identity between *C. elegans* and *G. pallida* (cloned based on gene model GPLIN_000654600) CAT-1a is 51.8%, similar positions 95. * - identical positions, : and . - similar positions.

gp_cat-1 MAQWLDSFVQKYRSNRRMLLSIVYVALFLDNMLLTTVVPIIPEYLLRLQHPNSTDMLLNN 60

ce_cat-1a -MSYILDWISTYRNNRKILLLIVYIALFLDNMLLTTVVPIIPEYLLRMEHPNETDILFSH 59

ce_cat-1b -MSYILDWISTYRNNRKILLLIVY------------------------------------ 23

.:: .::..**.**::** ***

gp_cat-1 VFSEQELAAPPATPFPPSDKVRVKRQTIGWD-SAEWENSLKNDETEQKKPRRFKGKALVA 119

ce_cat-1a NSQK-----------------RVKR---QWEDDDSWDVPLKLGNNADI---DWDENPIGP 96

ce_cat-1b -----------------------------------------------I---DWDENPIGP 33

:. : :

gp_cat-1 GGMRGGRVRTTMPPPLDMDDDYEALPDEFSIESPIDELDLPSDRSKSMGRRRTSKRPNST 179

ce_cat-1a VGRSERRKES-------------------------DRKN-HNSKNREYNDRRKYMSPAAK 130

ce_cat-1b VGRSERRKES-------------------------DRKN-HNSKNREYNDRRKYMSPAAK 67

* * .: *. : ..:.:. **. * :.

gp_cat-1 TSSQSSAERQQIAAAAFREVRHRTLTEENVHVGLMFGSKALVQLLANPLVGPLTNKIGYT 239

ce_cat-1a KP--PVETEEPAVRTISEEERHKLLASENVHVGLMFGSKALVQLLVNPWIGPLTNRIGYT 188

ce_cat-1b KP--PVETEEPAVRTISEEERHKLLASENVHVGLMFGSKALVQLLVNPWIGPLTNRIGYT 125

. .: . : .* **: *:.******************.** :*****:****

gp_cat-1 MPMFAGFVIMFLSTLLFAFGTSFITLWLARSLQGIGSACTSTSGMGMLAQAYPDDAERGS 299

ce_cat-1a MPMFGGFVIMFCSTILFAFGDSYFTLWLARALQGVGSACTSTSGMGMLAQAYPDDLERGS 248

ce_cat-1b MPMFGGFVIMFCSTILFAFGDSYFTLWLARALQGVGSACTSTSGMGMLAQAYPDDLERGS 185

****.****** **:***** *::******:***:******************** ****

gp_cat-1 AMGIALGGLALGVLVGPPYGGMLYQWAGKELPFILLALLALLDGSLQFLLLQPKVDRGEP 359

ce_cat-1a AMGIALGGLALGVLVGPPYGGLLYQWSGKELPFVLLALLALFDGSIQFMVLQPKIDRGEP 308

ce_cat-1b AMGIALGGLALGVLVGPPYGGLLYQWSGKELPFVLLALLALFDGSIQFMVLQPKIDRGEP 245

*********************:****:******:*******:***:**::****:*****

gp_cat-1 EGTAIKELARDPYIIVAAGSITIGNLGIAMLEPSLPLWMMESWSANSIERGAAFLPASIS 419

ce_cat-1a EGSSIKQLAKDPYIIVAAGAITIGNLGIAMLEPSLPLWMMESWGANSLERGAAFLPASIS 368

ce_cat-1b EGSSIKQLAKDPYIIVAAGAITIGNLGIAMLEPSLPLWMMESWGANSLERGAAFLPASIS 305

**::**:**:*********:***********************.***:************

gp_cat-1 YLIGTNIFGPLAYKIGRWLSSFIGLIVIGFCLIAIPSASSVFGLILPNFCMGFSIGMIDA 479

ce_cat-1a YLIGTNIFGPLAHRIGRWLSSFIGLVVIGFSLLSIPSATSVAGLIIPHALLGFSIGMIDA 428

ce_cat-1b YLIGTNIFGPLAHRIGRWLSSFIGLVVIGFSLLSIPSATSVAGLIIPHALLGFSIGMIDA 365

************::***********:****.*::****:** ***:*. :*********

gp_cat-1 SMFPMMGHIVDQRHVGVYGSIYAIADAAFCFAFFLGPFFSGPLVRTVGFPTMMYMIAVVN 539

ce_cat-1a SMFPLMGYLVDIRHVGVYGSIYAIADAAFCFAFALGPFFSGPLVKSLGFPTMMYIIAVIS 488

ce_cat-1b SMFPLMGYLVDIRHVGVYGSIYAIADAAFCFAFALGPFFSGPLVKSLGFPTMMYIIAVIS 425

****:**::** ********************* **********:::*******:***:.

gp_cat-1 FCYAPLMFFLRVLPAKVPEEQAIVVHADVSSGGRPPLVQRQQSVVRHHLYEKIEGAVDFQ 599

ce_cat-1a FLYAPLMFLLKNPPVLIEPTPQTQ---------SVELRQNGDSRVTNENYERIEGMNIAG 539

ce_cat-1b FLYAPLMFLLKNPPVLIEPTPQTQ---------SVELRQNGDSRVTNENYERIEGMNIAG 476

* ******:*: *. : * *. :* * .. **:***

gp_cat-1 LDTGAAATSARNSHYNGYNFSSVWED 625

ce_cat-1a Q-------LQQNN---MYSATSAF 553

ce_cat-1b Q-------LQQNN---MYSATSAF 490

:*. *. :*.:
