# Supplementary material for: Identification and characterisation of serotonin signalling in the potato cyst nematode Globodera pallida reveals new targets for crop protection
Source: PLoS Pathog. 2020 Oct 2;16(10):e1008884. doi: 10.1371/journal.ppat.1008884 (PMC7556481; doi:10.1371/journal.ppat.1008884)
Supplement: S2 Fig — UNIPROT, CLUSTAL Omega program, accessed on 14/12/2014. Cloned Gp-TPH-1 is based on G. pallida gene model GPLIN_000790300. Identity across all sequences is 56.4%. Identical positions 308, similar positions 79. Identity between G. pallida TPH-1 and C. elegans TPH-1a is 63.7%. *—identical positions,: and.—similar positions. (DOCX) [file ppat.1008884.s002.docx]

**Supplementary Figure 2. Alignment of amino acid sequences of *G. pallida* (gp) and *C. elegans* (ce) TPH-1.** UNIPROT, CLUSTAL Omega program, accessed on 14/12/2014. Cloned Gp-TPH-1 is based on *G. pallida* gene model GPLIN_000790300. Identity across all sequences is 56.4%. Identical positions 308, similar positions 79. Identity between *G. pallida* TPH-1 and *C. elegans* TPH-1a is 63.7%. * - identical positions, : and . - similar positions.

gp_tph-1 ------MASGMKFLYYNQKTPARRTMST---SMSEHRLEELKQRFRRSGSLGIPFFPEGE 51

ce_tph-1_a MDSLFQMASAMKFQYYSKKAAGKT-MSNSVSMSSDNRMEDFKRRFRRSGSLGIPFVPEED 59

ce_tph-1_b ------------------------------------------------------------

gp_tph-1 DGMELKKELTI---AETDESMEEVGSALLTVVVRAQRGIPTLTKTLSTLQRGVQLKHFES 108

ce_tph-1_a VKQLFTPTRTVRREASIREGDEEEGVQILTIIVKSSRVSEDISKMIANLPDHTRIKHLET 119

ce_tph-1_b --------------------------------------------MIANLPDHTRIKHLET 16

::.* .::**:*:

gp_tph-1 RDPKDGQPNELEVLLELELEGGINGNDVLMVLQRAGFLVHELTRTFAPKGAV-EPSDPGS 167

ce_tph-1_a RDSQDGSSKTMDVLLEIELFHYG-KQEAMDLMRLNGLDVHEVSSTIRPTAIKEQYTEPGS 178

ce_tph-1_b RDSQDGSSKTMDVLLEIELFHYG-KQEAMDLMRLNGLDVHEVSSTIRPTAIKEQYTEPGS 75

** :**. : ::****:** ::.: ::: *: ***:: *: *.. : ::***

gp_tph-1 ADALSGAPWFPKSIYDLDICSKRVIMYGAGLDAEHPGFKDEDYRRRRMMFAELALNYKMG 227

ce_tph-1_a DDATTGSEWFPKSIYDLDICAKRVIMYGAGLDADHPGFKDTEYRQRRMMFAELALNYKHG 238

ce_tph-1_b DDATTGSEWFPKSIYDLDICAKRVIMYGAGLDADHPGFKDTEYRQRRMMFAELALNYKHG 135

** :*: ************:************:****** :**:************* *

gp_tph-1 EPIPRVEFTECEKRTWTIIYRKLRELHGKYACQQFLDNFVLLERHCGYSANNIPQLEDVS 287

ce_tph-1_a EPIPRTEYTSSERKTWGIIYRKLRELHKKHACKQFLDNFELLERHCGYSENNIPQLEDIC 298

ce_tph-1_b EPIPRTEYTSSERKTWGIIYRKLRELHKKHACKQFLDNFELLERHCGYSENNIPQLEDIC 195

*****.*:*..*::** ********** *:**:****** ********* ********:.

gp_tph-1 RFLKAKTGFRVRPVAGYLSARDFLAGLAFRVFNCTQYIRHHADPFYTPEPDTVHELMGHM 347

ce_tph-1_a KFLKAKTGFRVRPVAGYLSARDFLAGLAYRVFFCTQYVRHHADPFYTPEPDTVHELMGHM 358

ce_tph-1_b KFLKAKTGFRVRPVAGYLSARDFLAGLAYRVFFCTQYVRHHADPFYTPEPDTVHELMGHM 255

:***************************:*** ****:**********************

gp_tph-1 ALFADPDFAQFSQEIGLASLGASEDDLRQLATLYFFSIEFGLCSSETNWGGGTEEVGLSN 407

ce_tph-1_a ALFADPDFAQFSQEIGLASLGASEEDLKKLATLYFFSIEFGLSSDDAADSP--------- 409

ce_tph-1_b ALFADPDFAQFSQEIGLASLGASEEDLKKLATLYFFSIEFGLSSDDAADSP--------- 306

************************:**::*************.*.:: .

gp_tph-1 GRCTARKSDKKSKFKIYGAGLLSSAGELQHAVEGNSEILRFDPDRVVQQECLITTFQTAY 467

ce_tph-1_a ---VKENGSNHERFKVYGAGLLSSAGELQHAVEGSATIIRFDPDRVVEQECLITTFQSAY 466

ce_tph-1_b ---VKENGSNHERFKVYGAGLLSSAGELQHAVEGSATIIRFDPDRVVEQECLITTFQSAY 363

. .:..::.:**:******************.: *:********:*********:**

gp_tph-1 FYTRNFEEAQQKLRTFTSNMNRPFVVRYNAYTESVEVLNNKRSLMLAVNSLRSDINLLAA 527

ce_tph-1_a FYTRNFEEAQQKLRMFTNNMKRPFIVRYNPYTESVEVLNNSRSIMLAVNSLRSDINLLAG 526

ce_tph-1_b FYTRNFEEAQQKLRMFTNNMKRPFIVRYNPYTESVEVLNNSRSIMLAVNSLRSDINLLAG 423

************** **.**:***:**** **********.**:***************.

gp_tph-1 SLHNIL 533

ce_tph-1_a ALHYIL 532

ce_tph-1_b ALHYIL 429

:** **
