# Supplementary material for: Identification and characterisation of serotonin signalling in the potato cyst nematode Globodera pallida reveals new targets for crop protection
Source: PLoS Pathog. 2020 Oct 2;16(10):e1008884. doi: 10.1371/journal.ppat.1008884 (PMC7556481; doi:10.1371/journal.ppat.1008884)
Supplement: S3 Fig — Alignment of amino acid sequences of G. pallida (gp) and C. elegans (ce) SER-7. In the alignment shown '*' indicates identical amino acids, ':' and '.' indicate similar amino acids at each position. The identity between G. pallida SER-7 and C. elegans SER-7a is 33.2%. CLUSTAL O(1.2.1) multiple sequence alignment (10/09/2015). (DOCX) [file ppat.1008884.s003.docx]

**Supplementary Figure 3. Amino acid identity between the *C. elegans* (ce) and *G. pallida***

**(gp) serotonin receptor, SER-7**. Alignment of amino acid sequences of *G. pallida* (gp) and *C. elegans* (ce) SER-7. In the alignment shown '*' indicates identical amino acids, ':'

and '.' indicate similar amino acids at each position. The identity between *G. pallida* SER-7

and *C. elegans* SER-7a is 33.2%. CLUSTAL O(1.2.1) multiple sequence alignment (10/09/2015)

gp_ser-7 MVCRILLLFCIPSSFASSSSSGGPDPSQILERVDHSLFDAWPSTSTPWDTVLSNYSTPSV 60

ce_ser-7a -------------------MARAVNISPFASYTVVPITSAWPPDDLKADR---------- 31

ce_ser-7b -------------------MARAVNISPFASYTVVPITSAWPPDDLKADR---------- 31

ce_ser-7c -------------------MARAVNISPFASYTVVPITSAWPPDDLKADR---------- 31

: . : * : . . : .*** . *

**TM1**

gp_ser-7 STTTAADMAPIRTGPSTSLFHSRPMVALLITALVFVLIVATVLGNLMVCVAIALVRKLKA 120

ce_ser-7a -----VQLASFTT--------G----KALLAIAILAMIIMTTVGNALVCLAVLLVRKLK- 73

ce_ser-7b -----VQLASFTT--------G----KALLAIAILAMIIMTTVGNALVCLAVLLVRKLK- 73

ce_ser-7c -----VQLASFTT--------G----KALLAIAILAMIIMTTVGNALVCLAVLLVRKLK- 73

.::* : * . *:: ::.:*: *.:** :**:*: ******

**TM2**

**TM3**

gp_ser-7 QPANLLLVSLAVADFCVGLFVMPMAAVYLLEDKWPFGSLLCCFWVTADLTLCTASILNLC 180

ce_ser-7a HPQNFLLVSLAVADFFVGLVVMPLALIDLLFDKWPLGSTMCSVYTTSDLTLCTASIVNLC 133

ce_ser-7b HPQNFLLVSLAVADFFVGLVVMPLALIDLLFDKWPLGSTMCSVYTTSDLTLCTASIVNLC 133

ce_ser-7c HPQNFLLVSLAVADFFVGLVVMPLALIDLLFDKWPLGSTMCSVYTTSDLTLCTASIVNLC 133

:* *:********** ***.***:* : ** ****:** :*..:.*:*********:***

**TM4**

gp_ser-7 MISV**DR**LLA**V**TR**AL**RYLAIR**T**RRRICAVIGAVWVGSLVVSAVPLAL------VPFRGNGN 234

ce_ser-7a AISV**DRY**LV**I**SS**PL**RY**S**AKR**TT**KRIMMYIACVWIIAAIVSISSHIIANLLNDGTYVDDTG 193

ce_ser-7b AISV**DRY**LV**I**SS**PL**RY**S**AKR**TT**KRIMMYIACVWIIAAIVSISSHIIANLLNDGTYVDDTG 193

ce_ser-7c AISV**DRY**LV**I**SS**PL**RY**S**AKR**TT**KRIMMYIACVWIIAAIVSISSHIIANLLNDGTYVDDTG 193

***** *.:: *** * ** :** *..**: : :** : : :

**TM5**

gp_ser-7 VCQVSQNRFYQIIATSIAFWVPALIMVIVYVKLWNAAKKMQRQDRMVLRWLGVQCQRQQN 294

ce_ser-7a TCQVIPHFIYQSYATIISFYAPTFIMVILNIKIWRAAKRLAAQDRLMSHCNSVDASERPR 253

ce_ser-7b TCQVIPHFIYQSYATIISFYAPTFIMVILNIKIWRAAKRLAAQDRLMSHCNSVDASERPR 253

ce_ser-7c TCQVIPHFIYQSYATIISFYAPTFIMVILNIKIWRAAKRLAAQDRLMSHCNSVDASERPR 253

.*** . :** ** *:*:.*::****: :*:*.***:: ***:: : .*:...: .

gp_ser-7 TEPTTPKANGTIREMATNNSSPSLLANANARLMTALRPSPTAQQIQRHSIEHNNNNVTAE 354

ce_ser-7a --------NGSAETK-------DFLNEK-ETI-----------DVPKKERANSTNSRLFK 286

ce_ser-7b --------NGSAETK-------DFLNEK-ETI-----------DVPKKERANSTNSRLFK 286

ce_ser-7c --------NGSAETK-------DFLNEK-ETI-----------DVPKKERANSTNSRLFK 286

**: . .:* : : :: ::. ...*. :

**TM6**

gp_ser-7 LDK---PRPSACSMLAGAIRIPLLGSYSSTASSVVGKSQHIQHEDKARKTLGVMMSVFIC 411

ce_ser-7a LERKYLHRPSA---F-----------FSAAKGPL--IRQ**T**EKSECKARKTLGVIMSVFII 330

ce_ser-7b LERKYLHRPSA---F-----------FSAAKGPL--IRQ**T**EKSECKARKTLGVIMSVFII 330

ce_ser-7c LERKYLHRPSA---F-----------FSAAKGPL--IRQ**T**EKSECKARKTLGVIMSVFII 330

*:: **** : :*:: . : * : * ********:*****

**TM7**

gp_ser-7 C**W**MPF**F**ILALLKSQRIVYHVPTWLDSLAL**W**LG**Y**SNSML**NP**LIYCKYNREFRIPFREMICC 471

ce_ser-7a C**W**LPF**F**ILAIFKSFG--MWIPDWLDLLAL**W**LG**Y**SNSTL**NP**LIYCKYNKEFRIPFREMLAC 388

ce_ser-7b C**W**LPF**F**ILAIFKSFG--MWIPDWLDLLAL**W**LG**Y**SNSTL**NP**LIYCKYNKEFRIPFREMLAC 388

ce_ser-7c C**W**LPF**F**ILAIFKSFG--MWIPDWLDLLAL**W**LG**Y**SNSTL**NP**LIYCKYNKEFRIPFREMLAC 388

**:******::** :* *** ********** **********:*********:.*

gp_ser-7 RFATLQDAMRNESYYAKFGSPR**S**RNGRRLNSTASRNWPPT--ERNDGQKRDGAGGGNGKG 529

ce_ser-7a RCATLQTVMRQQSF**T**SRYGPPVRYRTQSS------SYRPLLSRRNDSHEASDV------- 435

ce_ser-7b RCATLQTVMRQQSF**T**SRYGPPV**S**-------------------RRNDSHEASDV------- 422

ce_ser-7c RCATLQTVMRQQSF**T**SRYGPPV-------------------------------------- 410

* **** .**::*: :::* *

gp_ser-7 RHSLAVGPVTADKRNSTPGALAN 552

ce_ser-7a -----------------------

ce_ser-7b -----------------------

ce_ser-7c -----------------------

.

Transmembrane domains

Dark grey PDZ motif

**In green** – **PKC** phosphorylation sites

**In pink** – **PKA** phosphorylation sites

Light grey – domain involved in G-protein coupling.

Highly conserved residues are in **blue**.

**In red** – amino acids conserved in biogenic amine binding.

**NP** – sequestration and desensitization.

Light blue – region that possibly contains splice variants.
