# Supplementary material for: Identification and characterisation of serotonin signalling in the potato cyst nematode Globodera pallida reveals new targets for crop protection
Source: PLoS Pathog. 2020 Oct 2;16(10):e1008884. doi: 10.1371/journal.ppat.1008884 (PMC7556481; doi:10.1371/journal.ppat.1008884)
Supplement: S4 Fig — The cloned G. pallida mod-1 is based on gene model GPLIN_001254300. In the alignment shown 'І' indicates identical amino acids, ':' and '.' indicate similar amino acids at each position. The identity between G. pallida MOD-1 and C. elegans MOD-1a is 46.1%. There are 55.7% similar positions. (DOCX) [file ppat.1008884.s004.docx]

**Supplementary Figure 4. Alignment of amino acid sequences of *G. pallida* (Gpa) and *C. elegans* (Ce) MOD-1.** The cloned *G. pallida* *mod-1* is based on gene model GPLIN_001254300. In the alignment shown 'І' indicates identical amino acids, ':' and '.' indicate similar amino acids at each position. The identity between *G. pallida* MOD-1 and *C. elegans* MOD-1a is 46.1%. There are 55.7% similar positions.

EMBOSS Needle pairwise sequence alignment

CeMOD-1a --------MKFIPEITLLLLLFVH---STQAKGKRRKCPEGAWSEGKIMN 39

...:| |||||.|| ..:|..:|:||| |:.||.||:.

GpaMOD-1 MLCPTGRRRLLLP---LLLLLAVHFQLHRKAIARRQKCP-GSLSEDKILK 46

CeMOD-1a TIMSNYTKMLPDA---EDSVQVNIEIHVQDMGSLNEISSDFEIDILFTQL 86

.:..||||.|||: ::||.|.:|:||||||||||||:|||||||||||

GpaMOD-1 EMFRNYTKRLPDSDHKDESVHVEVEMHVQDMGSLNEISADFEIDILFTQL 96

CeMOD-1a WHDSALSFAHLPA-CKRNITMETRLLPKIWSPNTCMINSKRTTVHASPSE 135

|||.:|||.:|.: |.||||||:|.|..||:||||:||:|||:|||||::

GpaMOD-1 WHDPSLSFVNLSSECVRNITMESRYLKDIWTPNTCIINAKRTSVHASPAD 146

CeMOD-1a NVMVILYENGTVWINHRLSVKSPCNLDLRQFPFDTQTCILIFESYSHNSE 185

|:|.|||||||:|.|||||||:||.||||.||||||||.|:.||||||:|

GpaMOD-1 NIMFILYENGTIWTNHRLSVKAPCALDLRTFPFDTQTCELLLESYSHNNE 196

CeMOD-1a EVELHWMEEAVTLMKPIQLPDFDMVHYSTKKETLLYPNGYWDQLQVTFTF 235

||.||||.|.:|:|||||||||||:.::||:.::||||||||:||..|||

GpaMOD-1 EVTLHWMAEPITMMKPIQLPDFDMIQFATKRVSVLYPNGYWDELQARFTF 246

CeMOD-1a KRRYGFYIIQAYVPTYLTIIVSWVSFCMEPKALPARTTVGISSLLALTFQ 285

||||||||:|||||||||||||||:|||||||||||||||:|||||||.|

GpaMOD-1 KRRYGFYILQAYVPTYLTIIVSWVAFCMEPKALPARTTVGVSSLLALTLQ 296

CeMOD-1a FGNILKNLPRVSYVKAMDVWMLGCISFVFGTMVELAFVCYISRCQNSVRN 335

||||.|||||||||||||||||||||||||||:|||.||||:|.|:.:|:

GpaMOD-1 FGNIHKNLPRVSYVKAMDVWMLGCISFVFGTMIELAVVCYITRRQSMMRS 346

CeMOD-1a AERRRERMRN------SQVWANGSCRTRSNGYANG--------------- 364

...||...|. ||.:|:.:.|:.::..|.|

GpaMOD-1 RLARRSSSRGDSLSMYSQHFAHSNRRSFASSCALGDEPPGENTPKKMNYC 396

CeMOD-1a -----------------GSVISHYHPTSNG------NGNNNRH------- 384

.||..|..|..|| ||...|.

GpaMOD-1 PEATSFRLLATQRGSRPSSVRLHTLPVRNGTIPSSTNGAQRRRTSNSSNC 446

CeMOD-1a ----------------------------------------DTPQVTGRGS 394

.|....|.|.

GpaMOD-1 GGIFPLARRRLKRRSEIDRDVHAGDKSPAIFHPIKQKDDISTTAEVGTGD 496

CeMOD-1a L------HRN----GPPSPLNLQMTTFDSE-------IPLTFDQL----- 422

: |.: .||: |...|.|: ||:...|.

GpaMOD-1 ICIAVPTHTHCAAASPPA----QRRAFRSQNGSVPEGIPIDKAQTSAGEE 542

CeMOD-1a ----PVSMESDRPLIEEMRSTSPPP-PSGCLAR----------------- 450

.|...|...:.|||....|.| |...:.|

GpaMOD-1 RHNNSVDKRSANAIPEEMFPFIPSPMPMPSIGRPLRPSLSSRWRSFGDEE 592

CeMOD-1a --------FHPEAVDKFSIVAFPLAFTMFNLVYWWHYLS--QTFDQNYQ- 489

..||::||.|||.|||.||:|||:|||:||| ..||.:..

GpaMOD-1 EEEENRLIIQPESIDKLSIVVFPLTFTLFNLIYWWYYLSLGSQFDWDNDG 642

CeMOD-1a -------------- 489

GpaMOD-1 TLVNQHNDDRPNKQ 656
