# Supplementary material for: Identification and characterisation of serotonin signalling in the potato cyst nematode Globodera pallida reveals new targets for crop protection
Source: PLoS Pathog. 2020 Oct 2;16(10):e1008884. doi: 10.1371/journal.ppat.1008884 (PMC7556481; doi:10.1371/journal.ppat.1008884)
Supplement: Attachment — Submitted filename: CRISFORD ET AL RESPONSE TO REVIEWERS.docx [file ppat.1008884.s006.docx]

**PPATHOGENS-D-20-00790 Decision letter 2 6 2020 Major revisions**

**CRISFORD ET AL RESPONSE TO REVIEWERS**

Thank you for providing us with the opportunity to submit a significantly revised manuscript. The response to reviewers and actions taken are described in detail below. Each reviewer comment is followed by an itemised RESPONSE in the listing below:

**Part I - Summary**
Reviewer #1: Manuscript PPATHOGENS-D-20-00790 describes the characterisation of components of the serotonin signalling pathway in the potato cyst nematode G. pallida. The authors adopt a novel approach combining chemical genetics and “model-hopping” to describe in considerable detail a pathway that has remained largely unexplored in any plant-parasitic nematode. Further, they demonstrate that disruption of serotonin signalling has a major impact on several aspects of the parasite life style “pre-biotrophy” (i.e. hatching and host entry). The manuscript is well written, the results unambiguous and well described (if a little repetitive), and the conclusions well justified by the data. I recommend the manuscript is accepted for publication following the most minor of revisions.

RESPONSE. We thank reviewer #1 for these positive comments.

Reviewer #2: The authors present their analysis of the serotonergic nervous system of the potato cyst nematode Globodera pallida. They accomplish this through a combination of pharmacological analysis and rescue of mutations in homologous genes of C. elegans. They find that G. pallida uses a similar/identical molecular pathway to regulate the serotonergic system as found in C. elegans. While the findings do not uncover novel mechanisms specific to serotonin signaling in PPNs and the authors have already demonstrated the utility of using C. elegans as a system to investigate gene function of PPNs (Costa et al., 2009), they do make a convincing argument that investigation of this component of the PPN nervous system will lead to insight into parasitism. Overall, I find this a very nice contribution to the field that will be of broad interest.

Generally, I find the claims properly placed in the context of previous literature and well-supported by the data. The only large concern I have is in relation to their conclusions regarding nematode movement and host entry (detailed below). This could be changed by focusing more on the stylet thrusting behavior. Also, while it would enhance the story to see expression (antibody or FISH) for the proteins or genes discussed, this is not absolutely needed for the particular claims. I also have relatively minor editorial suggestions. Overall, the manuscript is very well written.

RESPONSE. We thank reviewer #2 for the positive comments. In response to the 'large concern' please note that we do not make conclusions regarding nematode movement and host entry. Whilst we do report effects of reserpine on J2 movement, we make the key point in the abstract that the impact of reserpine on J2 host entry is due to an inhibition of stylet activity. We state, " reserpine… potently impairs the ability of the potato cyst nematode Globodera pallida to enter the host plant root. We show this is due to an inhibition of serotonergic signalling that is essential for activation of the stylet which is used to access the host root.".

Reviewer #3: In this manuscript, Crisford and colleagues showed that reserpine inhibits serotonergic signaling that is essential for nematode stylet activation and root invasion. Authors further identified the components of serotonin signaling pathway in PCN G. pallida. Since genetic transformation of PPN is not yet established, they functionally characterized serotonin signaling components (VMAT, TPH, SER-7, MOD-1) by complementation of corresponding C. elegans mutants. Finally, authors used chemical inhibitors to confirm the role of serotonin signaling components on parasitism and root invasion.

RESPONSE. The reviewer #3 raises no queries in this section.

**Part II – Major Issues: Key Experiments Required for Acceptance**

Reviewer #1: (No Response)

Reviewer #2: My main critique revolves around the conclusion tying serotonin to infection. While I generally agree with their conclusions regarding stylet thrusting, in the abstract the authors claim that impairment of host entry following exposure to reserpine is due to serotonergic signaling.

RESPONSE: We show that stylet thrusting, which is an essential behaviour for root invasion, is dependent on serotonin and impacted by reserpine. Therefore, our argument that serotonin signalling is required for root invasion holds.

From my reading this connection is based on pharmacological inhibition assays without the corresponding pharmacological rescues as were done for the stylet thrusting data (which are very convincing).

RESPONSE: This connection is based on the inhibition of stylet thrusting by reserpine which we unequivocally show, as the reviewer agrees, is due to a depletion of serotonin.

For example, the authors show that incubation in reserpine reduces the number of J2 that can infect the roots and the ability of J2s to move. They also show that exposure to methiothepin also causes a reduction in nematodes able to enter the root system. However, both compounds can act outside of serotonin transport and signaling.

RESPONSE: Yes, it is true that reserpine and methiothepin can affect biogenic amine signalling other than serotonin. We raise this in the discussion. But we clearly demonstrate that a behaviour that is critical for host invasion i.e. stylet thrusting is dependent on serotonin and is inhibited by reserpine and methiothepin. The text at lines 138-150 and at line 180-187 has been amended accordingly:

*"As reserpine is an inhibitor of the VMAT (14) (Fig 2A), which loads a range of biogenic amine neurotransmitters including serotonin and dopamine into their respective synaptic vesicular storage sites, the effect of reserpine on dispersal could be explained by depletion of any of these neurotransmitters."*

*"* *Our observations do not provide a definition of the mechanism underpinning the inhibitory action of reserpine on J2 dispersal and this remains to be resolved. In contrast, it is clear that reserpine treatment leads to a sustained depletion of endogenous serotonin in neural circuits regulating stylet thrusting and this likely explains an inability of reserpine-treated nematodes to invade the host root."*

If reserpine is producing its effect through serotonin transport alone, then application of exogenous serotonin should rescue the reserpine phenotypes shown in Figure 1, similar to the stylet thrusting behaviors in Figure 2.

RESPONSE: It is not possible to conduct the experiments for serotonin rescue as the reviewer suggests: Applying serotonin to J2s causes causes coiling (our unpublished observations) and limits J2 movement.

Along these lines, the authors conclude that “consistent with previous reports that monoaminergic transmission, specifically that involving serotonin, is required for motility of plant-parasitic nematodes” They cite a review article by the senior author and an article by Masler et al as support. However, the Masler article found that exposure to serotonin caused a reduction in movement in both H. glycines and M. incognita (in contrast to McClure and Mende cited in the review). Horvitz et al found a similar inhibition of movement in response to exogenous serotonin application in C. elegans. This suggests that serotonin is inhibitory to movement. I would then expect reserpine to increase movement of G. pallida. If their conclusions are correct, then exposure to serotonin alone should increase the motility of J2s or at least have no effect on mobility. An alternative explanation is that reserpine is affecting multiple circuits in G. pallida (for example, dopamine). Either the authors should conduct these additional experiments or change the writing to focus on the stylet thrusting behavior.

RESPONSE. Yes, serotonin is inhibitory to nematode motility and therefore there is not a straightforward explanation for the effect of reserpine in the dispersal experiments. We have not established how reserpine affects dispersal and did not comment on this in the manuscript. We have now amended the text at line 181 to clarify that the mechanism underpinning the effect on dispersal is not resolved.

Reviewer #3: Overall, I am supportive of this interesting study as it provides a new approach to control PPN. However, I have some concerns regarding the data presentation and interpretation.
Figure 1: I am not sure why authors used hairy root for invasion assays? The results can be misleading. Why not use root system derived from internode cuttings?

RESPONSE: We developed hairy roots as an appropriate assay system and established that nematode development occurs within expected parameters (Urwin *et al*., 1995). In our hands, when care is taken to use matched, well-growing roots, they have proved to be reliable, in multiple published studies (eg. Atkinson *et al.,* 1996; Liu *et al*., 2005; Eves-van den Akker *et al*., 2014). Urwin *et al*. (1995) Engineered oryzacystatin-I expressed in transgenic hairy roots confers resistance to *Globodera pallida*. *Plant Journal* 8:121-131. Liu *et al.* (2005) The production of synthetic chemodisruptive peptides *in planta* disrupts the establishment of cyst nematodes. *Plant Biotechnology Journal* (2005) 3:487–496.Eves-van den Akker *et al*. (2014) Identification and characterisation of a hyper-variable apoplastic effector gene family of the potato cyst nematodes. *PLoS Pathogens* 10(9): e1004391.)

I also understood that invasion assays were performed in two independent replicates. This is unacceptable. At least, three independent experiments should be performed. This is valid for the entire manuscript.

RESPONSE: The n number for the root invasion assays throughout the manuscript is 20 individual plants and there were 2 independent experiments for each compound tested. The data are consistent and provide a clear result. Moreover, we show that 3 compounds that block serotonin signalling reserpine, CPA and methiothepin all block root invasion.

- Why to use different concentrations for figure 1A (100 uM) and 1B (50 uM)? It might be interesting to test different concentrations of reserpine for their effect on invasion.

RESPONSE: It would be interesting, but we would argue not necessary for this submission.

- Serotonin signaling components seem to be well conserved across different species of nematodes. So, I suggest performing invasion assays with different species of PPNs. This should support its role to control a broad of PPN.

RESPONSE: This would be interesting, but we would argue not essential for this manuscript.

Figure 2A: I suggest drawing an arrow in synaptic cleft pointing towards re-uptake of serotonin by its transporter. This should facilitate readers.

RESPONSE: Thank you for this suggestion. We have added an arrow to indicate the transport of serotonin from the cleft into the terminal.

Figure 3: Authors have performed ANOVA followed by Bonferroni’s multiple comparisons. However, only selective differences have been shown through asterisks. Can you please provide a lettering showing how different genotypes differ from each other? This is important to validate the claims that pharyngeal pumping movements were restored to wild type levels (N2) in complementation lines (Line 201). At the moment I just see that complementation lines were different from mutants (cat-1). This is valid for rest of the figures.

RESPONSE: The reviewer is asking us to provide the statistical evidence on the figures that the pharyngeal pumping rate of the transgenic rescue lines is not significantly different from wild type i.e. that the rescue is complete rather than partial. Thank you for encouraging us to include this more detailed representation of the statistical analysis. In our view it makes the figures rather cluttered to add statistical comparisons for all the experimental groups. Therefore to accommodate this reviewer's request we have added a supplementary file with all the statistical comparisons and also indicated in the figure legends the statistical analyses relating to the comparisons between the wild-type and the rescue lines where relevant. In some cases, this shows that in the rescue lines there is small but statistically significant difference between the wild type and rescue lines suggesting that the rescue may be partial. Where this is the case, we have drawn the readers' attention to it in the figure legend and in the results text.

**Part III – Minor Issues: Editorial and Data Presentation Modifications**

Reviewer #1: Below I provide a series for the authors consideration:

152 – “Reserpine potently blocked the stylet response to fluoxetine but not the response to serotonin (Fig 2D) consistent with an interpretation in which the response to fluoxetine requires the presence of correctly stored vesicular serotonin which is depleted by the VMAT-blocking action of reserpine (see Fig 2A).”

A lovely set of controls


161 - “Thus, the inhibition of stylet thrusting by reserpine is sustained for at least 22 h following its removal.”

An interesting result, and a shame the full duration of the effect wasn’t delineated/predicted.

RESPONSE: Lack of recovery after 22 hours treatment with reserpine indicated an enduring effect. Agreed, it would be interesting to extend this and determine the full duration but we would argue this isn't necessary for this submission.

Figure 2A
Consider improving the quality, clarity, and quantity of information in this panel.

RESPONSE: The explanation in panel A has been edited for clarity as follows:

**"A.** The cartoon provides a rationale for the differential effect of reserpine on the response to serotonin and fluoxetine. It depicts a serotonergic synapse, with the pre-synapse as the site of serotonin synthesis and release and the post-synapse which harbours serotonin receptors. In mammalian systems reserpine acts presynaptically to deplete the storage of the neurotransmitter serotonin (shown as open red circles) by preventing its uptake into vesicles (shown as solid red circles) by inhibition of the vesicular monoamine transporter, VMAT, present on the vesicle membrane (14). This prevents storage of serotonin and results in a lack of serotonin presynaptically and an inability of the presynaptic terminal to release serotonin. Fluoxetine (Prozac®) on the other hand blocks the plasma membrane serotonin transporter, SERT. This prevents the reuptake of serotonin into the presynaptic terminal following its release. Thus, fluoxetine increases synaptic levels of serotonin and in this way fluoxetine can act indirectly to stimulate transmission at the serotonergic synapse. This effect of fluoxetine is susceptible to block by reserpine as it requires endogenous serotonin to elicit its effect. However, the response to exogenous serotonin circumvents reserpine inhibition as it acts directly on the postsynaptic receptors."

Table 1 For gene absence, I suggest the authors additionally check the genome of the related species G. rostochiensis because it is a more faithful representation of the gene content of Globodera.

RESPONSE: In light of more in-depth analysis we have amended Table 1 to include *G. rostochienesis* and also added information to indicate that the “missing genes” are likely present in *G. pallida*, but were either absent from the genome, or their coding region incorrectly annotated.

Reviewer #2: It isn’t clear why a pan-neuronal promoter was used for most of the rescues except mod-1. While the data clearly shows rescue, it would be more convincing if endogenous promoters were used. I don’t think this experiment is necessary, but it would be valuable to include an explanation for the use of a pan-neuronal promoter and possible implications for data interpretation in the conclusion.

RESPONSE: We note the reviewer believes native rescue is not necessary. By way of explanation of our choice of promoter we have included this text in the methods Line 680: "This has the advantage of circumventing the need to clone the predicted native promoter for each gene of interest with the caveat that there will be ectopic expression in non-serotonergic circuits."

The Author Summary is primarily about reserpine and its botanical origin. This makes me think that the article will be about snakeroot and reserpine; however, reserpine is only the lead to the story and snakeroot is not featured at all. I would recommend rewriting to focus on PPNs and their behavior/neurobiology.

RESPONSE: We disagree. We think this context provides an engaging angle to a non-expert audience and the summary clearly states we have provided insight into the role of serotonin in PPN parasitic behaviour.

There are occasional changes between British and American spelling (neurone vs neuron). I’m guessing the copy editor will find these, but thought I’d mention.

RESPONSE: There are 3 instances of 'neurone'. This has now been changed to 'neuron'.

Similarly, the authors move between nematodes and “worms.” I recommend sticking to nematodes or J2s.

RESPONSE: This is not unusual for *C. elegans* parlance *cf* Wormbase. Nonetheless, we have made this change throughout the manuscript.

Line 139: Seems to be missing a noun.

RESPONSE: Thank you for pointing this out- corrected.

Line 251: Using a behavioral assay does not directly validate the ability of CPA to block TPH-1 expression. This could be done by measuring 5-HT levels. Perhaps change to “As an inhibitor of TPH, we predicted that CPA would inhibit pumping in *C. elegans*…”

RESPONSE: Thank you for this suggestion which we have used.

Line 302-303: What is meant by bona fide serotonin receptor? Does this mean that serotonin has the greatest affinity for it compared to other biogenic amines? If so, then wouldn't expression in COS cells and e-phys studies be important? I recommend toning this writing down.

RESPONSE: 'bona fide' has been removed.

Line 448: I’m not sure what a ‘model-hopping’ approach is. When I Googled this it came up with electron transport in solid materials.

RESPONSE: It means using *C. elegans* as model system to define the function of genes of interest from less experimentally tractable organisms of interest. An explanation has been included line 486-488:

" *i.e. we used C. elegans as model system to define the function of the G. pallida genes of interest through the generation of C. elegans transgenics expressing the G. pallida genes*."

Lines 518: What is the goal of drying cysts and treating with 0.1% malachite green? Is there a reference for this?

RESPONSE: The cysts were not specifically dried for use in the experiments. Cysts of *Globodera* species are routinely extracted from air-dried soil to facilitate separation by floatation and are then stored dry as they remain stable and viable in this condition for many years. The treatment of cysts with malachite green is an additional sterilisation step to help reduce bacterial and fungal contamination during hatching. The relevant wording on lines 559-560 has been amended for clarity.

The methods for the dispersal assay state there were concentric circles. I’m not sure I understand this. If it’s like I envision, then why wasn’t data shown for how far away from the origin the nematodes travelled in Figure 1B? I think this would be valuable rather than just how many left the origin.

RESPONSE: We extracted the key message for the purpose of the manuscript i.e. that reserpine inhibits dispersal However, in response to the reviewer's request we have amended Fig 1B and the results text line 127- 144 to provide a more detailed account of our observations.

"*Therefore, we tested the effect of reserpine on G. pallida motility and stylet activity. For motility, we employed a dispersal assay in which J2s were placed in the centre of an agar arena, demarcated with six concentric rings, with the central origin labelled '0' and the outermost ring numbered '5'. The number of J2s present in each concentric ring after a given period of time was scored. Although J2s were still moving after 18 h pre-exposure to reserpine (50 µM) their dispersal was significantly reduced. After 1 h in the agar arena 27.8 ± 2.0% of J2s were at the origin compared to 63.4 ± 3.6% of the reserpine treatment group (n=10 plates for each experimental group, from 2 independent repeats). This differential was sustained after the J2s had been on the agar arena for 2 h (Fig 1B).*"

Fig 1B amended shown below:

Lines 619-621: Are the primers used for amplification the ones listed in the table below. It wasn’t clear. Are the ATGs underlined in the table the start codons?

RESPONSE: Yes. The forward primer for pallida MOD-1 was just upstream in the 5’ UTR and so did not include the start codon. This has now been clarified in the text (lines 666-667).

Did the final plasmid injected include a 3’ UTR derived from C. elegans or elsewhere? Did it include an artificial intron? These are often helpful in C. elegans expression. If the pan-neuronal promoter was used because the endogenous ones didn’t work, perhaps this is the reason.

RESPONSE: The final expression vector contains 3’UTR terminator sequences corresponding to 3’UTR of the muscle myosin *C. elegans* gene *unc-54*, as well an optimized *C. elegans* intron to enhance expression. This information is added at line 709-712.

Provide a citation or source for pWormGate.

RESPONSE: This is the source for the pWormGate vectors and it has been added to the methods line 692: https://www.sciencedirect.com/science/article/pii/S0378111905003100?via%3Dihub

Line 678: Were these one day old adult hermaphrodites?

RESPONSE: Yes they were. This detail has been added, line 705.

Figure 2A: I thought the cartoon was helpful. It may be useful to include the other compounds such as methiothepin and list the serotonin receptors on this.

RESPONSE: We think this might make the main message, which is about the differential effect of reserpine on serotonin and fluoxetine, less clear and would prefer not to further complicate this cartoon.

Figure 8D: This doesn’t look like a DIC image, more like standard brightfield. If it is, then DIC should be spelled out.

RESPONSE: It is not DIC. This has now been removed from the legend.

Reviewer #3: What does n indicate in figure legends? Is this pooling of data points across the replicates? This should be clarified.

RESPONSE: 'n' represents the pooling of replicates. Each experiment was repeated on at least 3 independent occasions unless stated otherwise. This detail has been added to the methods, line 786-788.

I also recommend plotting of graphs in a way showing spread of data as in Figure 8 A and 8B.

RESPONSE: This has been done for all the bar graphs but not for the dose-response curves where it would result in a rather cluttered and confusing presentation.
